# Supplementary material for: Tuning the hysteresis of a metal-insulator transition via lattice compatibility
Source: Nat Commun. 2020 Jul 15;11:3539. doi: 10.1038/s41467-020-17351-w (PMC7363867; doi:10.1038/s41467-020-17351-w)
Supplement: Supplementary file 1 — Supplementary Information [file 41467_2020_17351_MOESM1_ESM.pdf]

SUPPLEMENTARY INFORMATION

Tuning the Hysteresis of a Metal-Insulator Transition via Lattice Compatibility

Y. G. Liang et al.

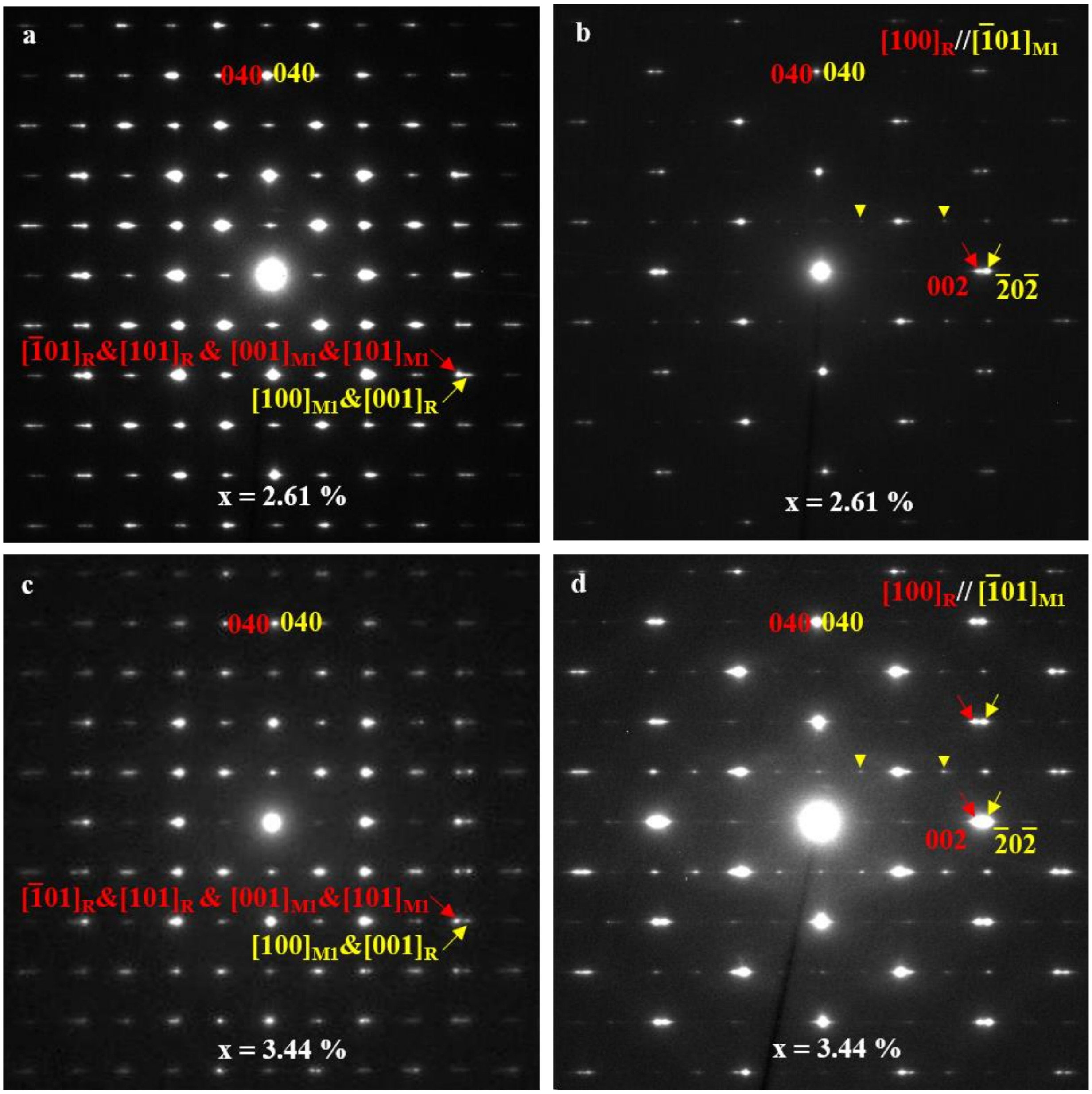

Supplementary Figure 1. **Structural analysis based on TEM images.** Typical selected area electron diffraction patterns (SAEDPs) taken from the epitaxial film of 2.61 % and 3.44 % W-doped $V_{1-x}W_xO_2$/*c*-Al_2O_3 heterostructure. Al_2O_3 substrate was orientated to [10-10] zone-axis (a, c), and [2-1-10] zone-axis (b, d), respectively. The arrows shown in (a) and (c) indicate that the splitting reflections can be indexed by multiple options belonging to the variants of M1 phase or R phase. The arrows in (b) and (d) indicate that the splitting reflections can be well indexed with the M1 phase and R phase.

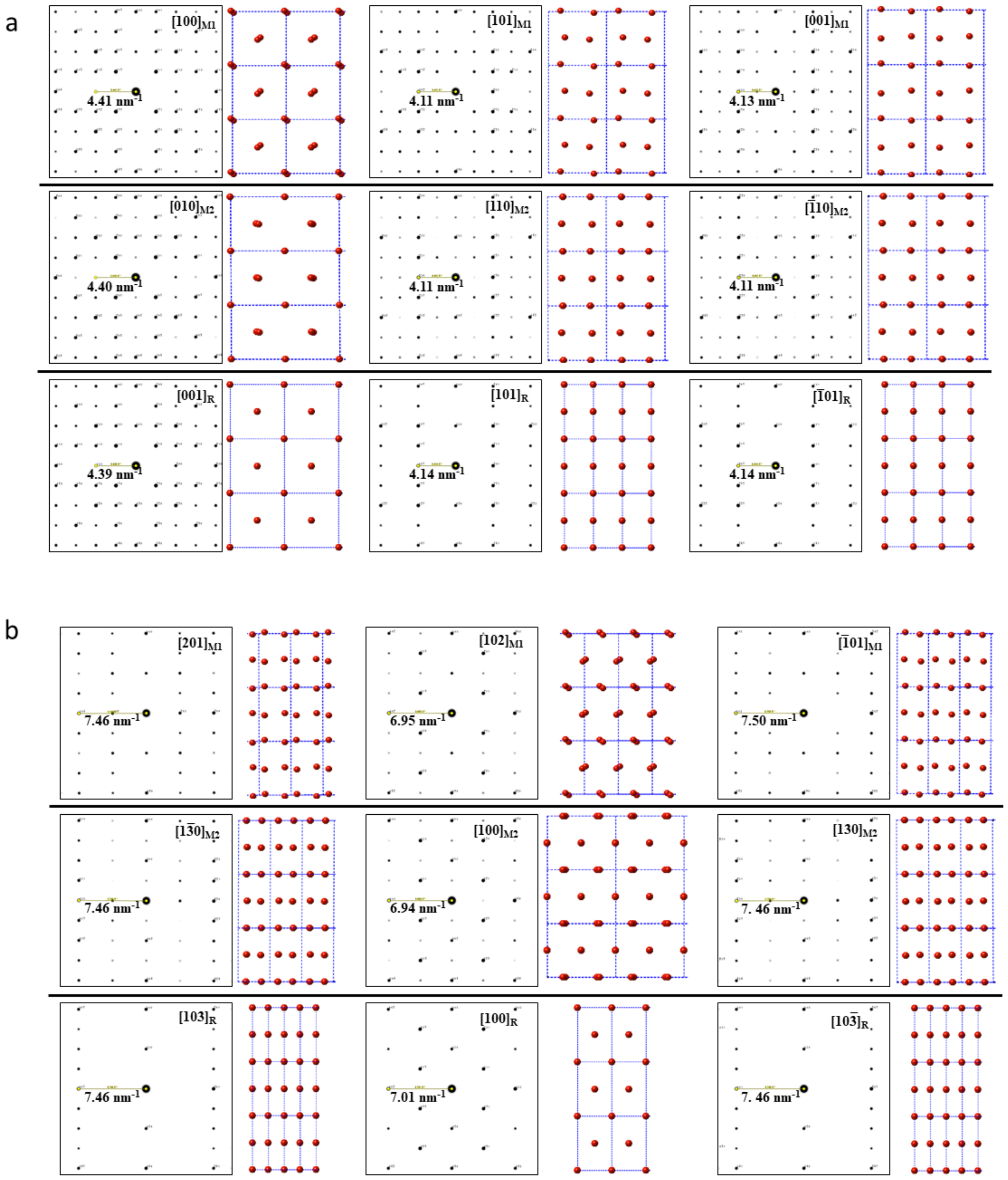

Supplementary Figure 2. **TEM result simulation.** Simulated electron diffraction patterns and the corresponding atomic projection of V atoms along the respective zone-axis. The patterns correspond to the possible variants of M1, M2 and R phases in the epitaxial $V_{1-x}W_xO_2/Al_2O_3$ film when Al_2O_3 is orientated to [10-10] zone-axis (a), and [2-1-10] zone-axis (b), respectively.

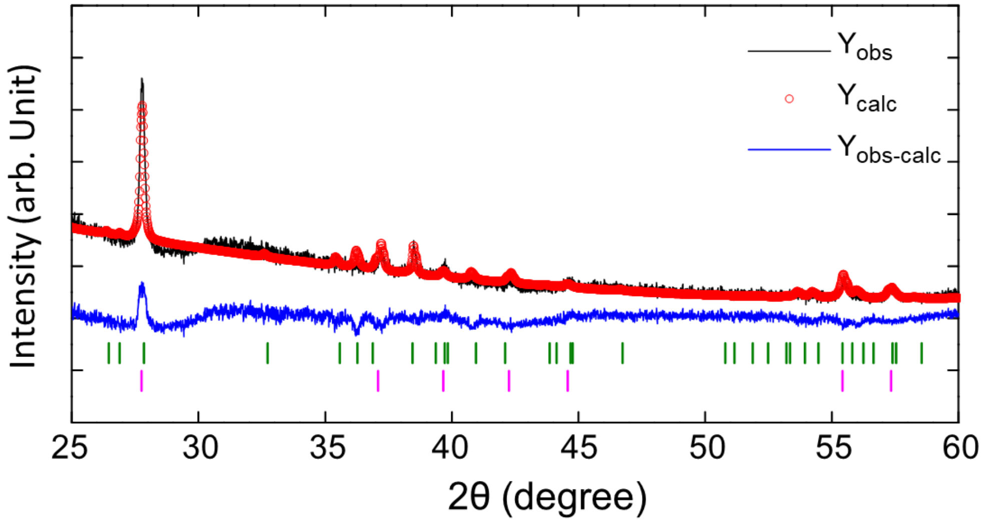

Supplementary Figure 3. XRD and Rietveld refinement example. XRD diffraction patterns of $V_{1-x}W_xO_2$ (x = 0.9 %) thin films and Rietveld refinement result. Measured diffraction pattern (Y_{obs}) and a calculated diffraction pattern (Y_{calc}) are represented as black lines and red circles, respectively. The difference between the measured and calculated diffraction patterns ($Y_{obs-calc}$) are indicated by a blue line. The olive and magenta bars provide the calculated Bragg peak positions of the M1 and the rutile phases, respectively.

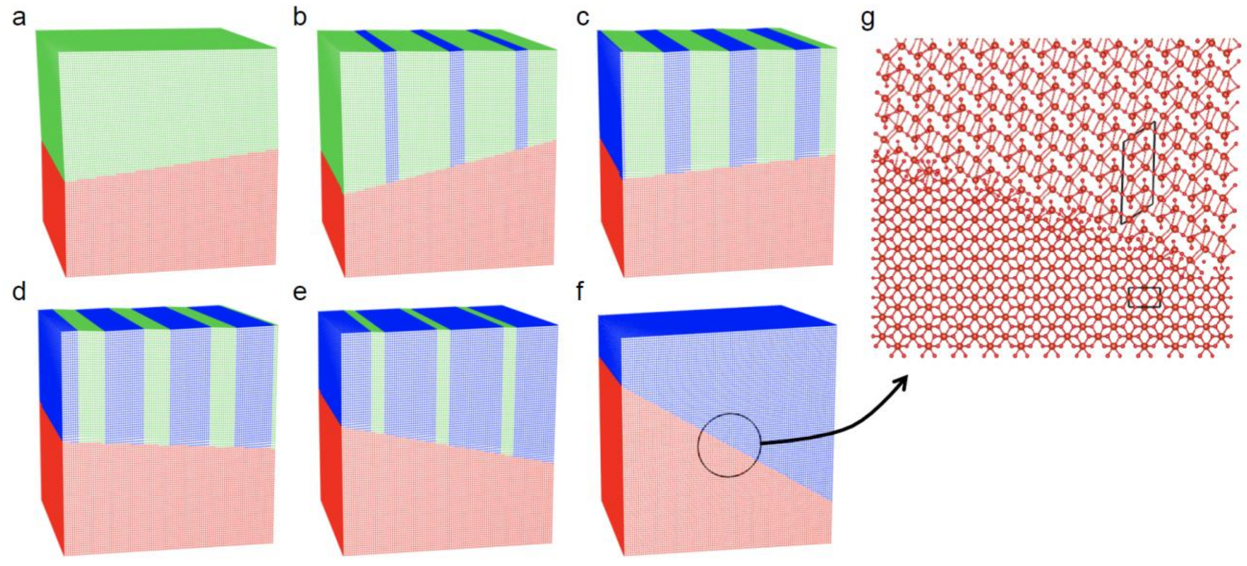

Supplementary Figure 4. Phase transformation simulation. Simulation of interfaces formed through the phase transformation in the $V_{1-x}W_xO_2$ system. (a)-(f) Supercompatible microstructures in $V_{1-x}W_xO_2$, determined from theory evaluated at the measured lattice parameters at x = 2.4 %. Front face is (100), red is tetragonal, and blue/green are two compound twinned monoclinic variants. Low energy interfaces are possible at all volume fractions of the twins and perfect unstressed interfaces are possible at volume fraction 0 or 1; (g) An atomistic view of a compatible interface (The unit cells of different variants).

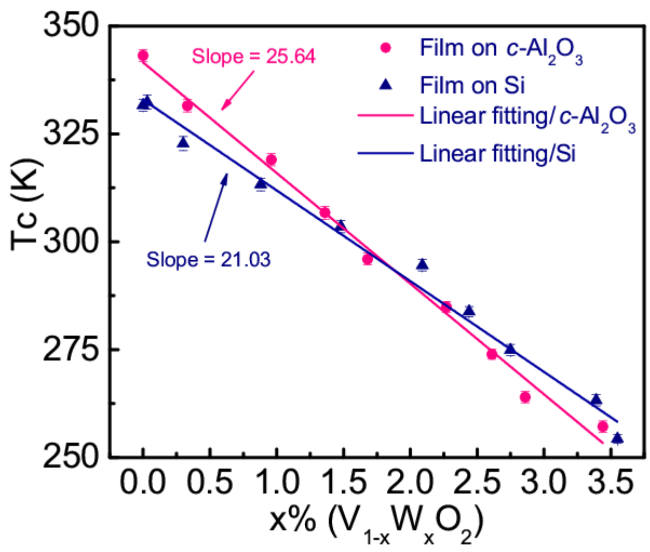

Supplementary Figure 5. Concentration dependence of the T_C. Dependence of the transition temperature (T_C) on W-concentration for samples on *c*-Al_2O_3 and Si. The error bars in the figure reflect the uncertainty of the temperature reading when repeating the thermal cycling between 200 K and 400 K at a scan speed of 1 K per minute for at least three times.

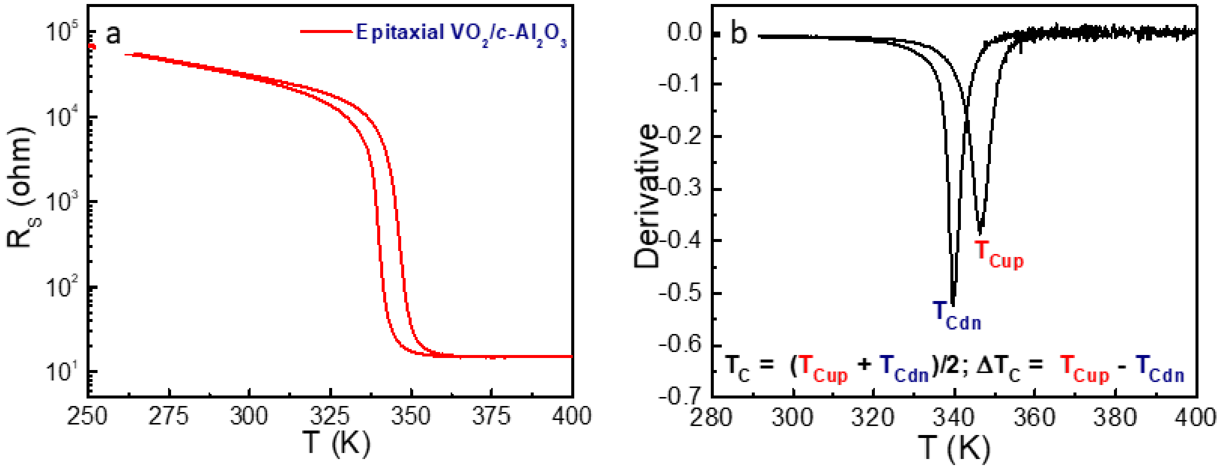

Supplementary Figure 6. An example of determining T_C and ΔT_C. (a) The R-T curve of pure VO_2 on *c*-Al_2O_3. (b) The first derivative plot for log(Rs)-T from which we extract the MIT properties: transition temperature (T_{Cup}, T_{Cdn}, and T_C) and transition hysteresis width ($\Delta T_C = T_{Cup} - T_{Cdn}$).

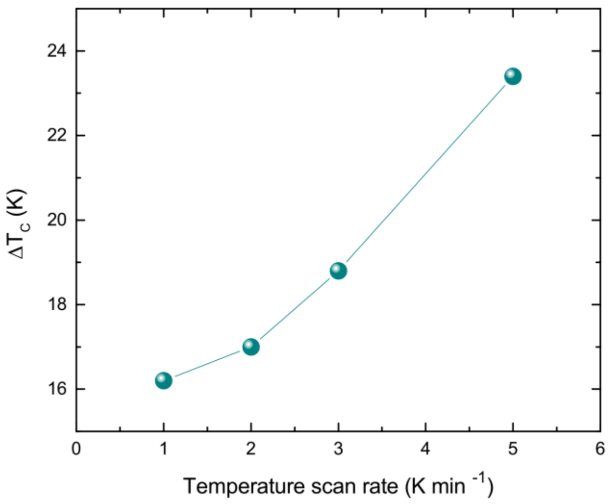

Supplementary Figure 7. Scan rate dependence of ΔT_C. Hysteresis width measured at different scan rates for a lightly substituted $V_{1-x}W_xO_2$ strip (x = 0.9 %) fabricated on a Si substrate. The scan rate dependence of the measured hysteresis width is caused by the latent heat associated with the first order transition. Therefore, it is important to keep the scan rate low and consistent for all measurements. In this study, a temperature scan rate of 1.0 K per minute was adopted for measurements on different sample strips.

x (%)	T (K)	% Phases		Lattice constant-Monoclinic Phase					Lattice constant-Tetragonal phase		
		%Mono	%Tetra	a (Å)	b (Å)	c (Å)	beta (deg.)	volume (Å^3)	a (Å)	c (Å)	volume (Å^3)
0	**300**	100	0	5.8751(0.0073)	4.5217(0.0021)	5.4861(0.0022)	124.359(0.016)	120.313(0.082)			
0.1(0.1)	**300**	100	0	5.8763(0.0015)	4.5221(0.0021)	5.4920(0.0022)	124.279(0.016)	120.591(0.083)			
0.3(0.1)	**300**	100	0	5.8999(0.0014)	4.5225(0.0020)	5.5037(0.0021)	124.535(0.015)	120.973(0.079)			
0.9(0.1)	**300**	65.6(0.15)	34.4(0.15)	5.9201(0.0016)	4.5208(0.0019)	5.5105(0.0023)	124.634(0.019)	121.347(0.084)	4.5424(0.0011)	2.86391(0.00070)	59.091(0.033)
1.5(0.1)	**300**	62.3(0.14)	37.7(0.14)	5.9356(0.0016)	4.5254(0.0020)	5.5367(0.0023)	125.029(0.019)	121.783(0.087)	4.5488(0.0012)	2.86120(0.00070)	59.203(0.034)
2.1(0.1)	**300**	63.50(0.1)	36.5(0.1)	6.0801(0.0019)	4.5311(0.0024)	5.5918(0.0025)	125.944(0.023)	124.72(0.10)	4.5457(0.0011)	2.85593(0.00057)	59.014(0.031)
2.4(0.1)	**300**	62.53(0.9)	37.47(0.9)	6.0652(0.0014)	4.5514(0.0017)	5.5878(0.0018)	125.748(0.017)	125.193(0.073)	4.55138(0.00090)	2.85959(0.00053)	59.237(0.026)
2.8(0.1)	**270**	65.90(0.79)	34.10(0.79)	6.1421(0.0015)	4.5784(0.0019)	5.6338(0.0020)	126.873(0.017)	126.736(0.080)	4.54883(0.00096)	2.85830(0.00060)	59.144(0.028)
3.4(0.1)	**270**	64.83(0.83)	35.17(0.83)	6.1607(0.0014)	4.5853(0.0018)	5.6325(0.0019)	126.798(0.015)	127.409(0.076)	4.5505(0.0010)	2.86554(0.00065)	59.336(0.029)
3.5(0.1)	**270**	48.78(0.97)	51.22(0.97)	6.2953(0.0015)	4.6135(0.0021)	5.7095(0.0020)	128.311(0.018)	130.114(0.087)	4.54333(0.00090)	2.86259(0.00059)	59.089(0.026)

Supplementary Table 1. Analysis of lattice parameters. Phase Fractional atomic-percentage of a $V_{1-x}W_xO_2$/Si ($0 \le x \le 3.5$ %) sample determined by Rietveld refinement. Data analysis was carried out using the TOPAS software. (Monoclinic phase: ICSD #74705, space group: $P2_1/c$; Tetragonal phase: ICSD #4110, space group: $P4_2/mnm$).
